# Multiple autologous tumor-infiltrating lymphocyte (LM103 infusion) therapy combined with immune checkpoint inhibitor induces repeated tumor regression in a patient with aggressive mucosal melanoma: a case report and literature review

**DOI:** 10.3389/fonc.2026.1789442

**Published:** 2026-04-23

**Authors:** Fenge Li, Yue Xu, Yupeng Wang, Ning Mu, Mei Liu, Weihong Feng, Yongming Xue, Shengnan Wu, Xinyi Wang, Gregory Lizee, Chunhua Ma

**Affiliations:** 1 Department of Oncology 5, Tianjin Union Medical Center, The First Affiliated Hospital of Nankai University, Nankai University, Tianjin, China; 2 Tianjin Cancer Institute of Integrative Traditional Chinese and Western Medicine, Tianjin Union Medical Center, The First Affiliated Hospital of Nankai University, Nankai University, Tianjin, China; 3 Department of Oncology, Tianjin Beichen Hospital, Tianjin, China; 4 Department of Basic Research, Suzhou Lanma Biotechnology Co., Suzhou, China; 5 Department of Melanoma Medical Oncology, The University of Texas MD Anderson Cancer Center, Houston, TX, United States; 6 Department of Immunology, The University of Texas MD Anderson Cancer Center, Houston, Houston, TX, United States

**Keywords:** clinical response, cytokine secretion, immune monitoring, multiple TIL therapy, tumor-infiltrating lymphocytes

## Abstract

Tumor-infiltrating lymphocyte (TIL) therapy was approved as a one-dose treatment indicated for melanoma patients who have progressed on anti-PD-1 therapy. However, the majority of patients show only short-duration responses, highlighting the urgent need to explore multiple cycles of TIL therapy and combination strategies. We report a case of a patient with aggressive stage IV nasal mucosal malignant melanoma with disease progression on multiple lines of immunotherapy who was enrolled in a clinical trial evaluating the safety and efficacy of autologous TIL therapy. Prior to TIL infusion, the patient underwent lymphodepletion with cyclophosphamide followed by fludarabine for 5 days. Approximately 24 hours later, the patient received an intravenous infusion of autologous TIL, followed by high-dose interleukin-2 for 6 days to support T-cell expansion and persistence. The patient achieved a partial response (PR) at 6 weeks and a complete response (CR) at 12 weeks post-infusion. However, tumor relapse occurred 7 months later. The patient then received a second TIL infusion, resulting in stable disease (SD) with transient shrinkage, but rapid regrowth occurred within 3 weeks. The patient subsequently received a third TIL infusion and achieved another PR within 2 weeks. Manageable adverse events were observed and resolved shortly after TIL treatments. A time-course study of peripheral blood cell subtyping and cytokine secretion demonstrated a long-term immune response. Longitudinal immune monitoring revealed sustained systemic immune activation. This case report shows that multiple autologous TIL therapies can induce repeated clinical and immunological responses in a heavily anti-PD-1-pretreated patient with advanced melanoma, underscoring the feasibility and therapeutic potential of multiple TIL treatments. **Clinical trial registration:**https://clinicaltrials.gov, identifier NCT06697665, NCT05941936, NCT05971589.

## Introduction

The five-year follow-up data of Lifileucel (TIL therapy) revealed that approximately 20% of patients with advanced melanoma survived beyond five years after TIL therapy—an unprecedented milestone in the treatment landscape of metastatic melanoma (1). As a form of adoptive cell transfer harnessing naturally occurring tumor-reactive T cells, TIL therapy has emerged as a promising adoptive cellular therapy for solid tumors, attracting growing global attention. With its recent FDA approval in the United States, researchers across Asia are accelerating efforts to develop and optimize TIL-based therapies, aiming to extend long-term survival benefits to broader patient populations (2–5).

The phase II C-144–01 trial demonstrated durable clinical outcomes in immune checkpoint inhibitor (ICI)-refractory melanoma patients treated with Lifileucel. Among 153 heavily pretreated patients (median of three prior systemic therapies), 28 completed five-year follow-up. The median overall survival (OS) was 57.8 months (cohort 2: 59.3 months; cohort 4: 57.6 months). The objective response rate (ORR) reached 31.4% (5.9% complete response [CR]; 25.5% partial response [PR]), with 79.3% (111/140) showing tumor burden reduction. Four patients maintained CR for up to 58.7 months, and 31.3% (15/48) of responders remained in response at five years. The estimated 5-year OS rate was 19.7%, providing compelling evidence of long-term survival potential even in ICI-resistant settings (1).

Despite these advances, approximately 70% of patients fail to respond to single-cycle TIL therapy. Consequently, combination approaches—particularly TIL plus PD-1 inhibitors—have shown enhanced anti-tumor activity in early studies (6–8). Moreover, for patients who initially respond but experience short progression-free survival (PFS), the role of repeated TIL infusions remains an open and critical question.

Here, we present a case of an Asian patient with aggressive metastatic nasal mucosal melanoma who had failed multiple anti-PD-1/CTLA-4 therapies. The patient received three sequential autologous TIL infusions combined with anti-PD-1 antibodies, resulting in rapid and clinically meaningful tumor regression in two of three TIL infusions. Serial analyses of peripheral T-cell subsets and cytokine profiles revealed dynamic systemic immune modulation correlating with clinical responses. This case underscores the feasibility and therapeutic potential of multiple TIL treatments and highlights the need for larger trials for defining optimal dosing, timing, and patient selection criteria.

## Methods

### Autologous TIL manufacturing

TIL products (LM103 infusion) were manufactured under Good Manufacturing Practice (GMP) guidelines by Suzhou Lanma Biotechnology Co., Ltd. Detailed expansion protocols have been previously described (5). Briefly, fresh tumor specimens were enzymatically dissociated and cultured in IL-2–containing medium. Expanded TILs were harvested, cryopreserved, and tested for sterility, viability, phenotype, and potency before release.

### Flow cytometry analysis

TIL phenotyping and peripheral immune monitoring used the following antibodies: FITC anti-human CD3 (BioLegend 300406), PE anti-human CD4 (317410), PerCP anti-human CD8 (344708), APC anti-human EpCAM (CD326; 369810), and PE/Cyanine7 anti-human TCRα/β (306720). For surface staining, ~1×10^6^ cells were washed in PBS and incubated with fluorochrome-conjugated antibodies (1:50 dilution) in FACS buffer (PBS + 0.5% FBS) for 30 min at 4 °C in the dark. After washing, samples were analyzed on a BD FACSCanto II cytometer. Data were processed using FlowJo v10 (BD Biosciences). Peripheral blood mononuclear cells (PBMCs) were isolated via RBC lysis (Solarbio R1010), then stained and analyzed identically to TILs (5).

### Enzyme-linked immunosorbent assay

Serum samples collected at predefined intervals were centrifuged and stored at -80 °C. Levels of IFN-γ (BioLegend 430107), TNF-α (Dakewe 1117202), IL-2 (BioLegend 431807), IL-6 (BioLegend 430507), IL-7 (Multi Sciences 70-EK107-96), IL-10 (BioLegend 430607), IL-13 (Multi Sciences 70-EK113-96), IL-21 (BioLegend 433807), and TGF-β1 (BioLegend 437707) were quantified using commercial ELISA kits according to the manufacturers’ instructions. Absorbance was measured at 450 nm with correction at 570 nm using a SpectraMax 190 Microplate Reader (Molecular Devices). Concentrations were calculated using the SoftMax Pro 7.1.2 software.

## Case presentation

A 59-year-old male with a history of smoking, type 2 diabetes (managed with subcutaneous degludec insulin), and well-controlled hypertension (on amlodipine besylate, max BP 150/100 mmHg) presented on July 19, 2021, with recurrent epistaxis. On July 20, 2021, he underwent endoscopic resection of a right nasal cavity mass under general anesthesia. Pathology confirmed mucosal malignant melanoma involving the right nasal cavity, round cushion, and inferior turbinate, with no bone, vascular, or neural invasion (max diameter ~0.7 cm). Immunohistochemistry showed: CK (pan)(−), vimentin (+++), S-100 (++), SOX-10 (+++), Melan-A (+), HMB-45 (+++), MiTF (++), Ki-67 (+) ~20%, p16 (+++), cyclinD1 (+++). Molecular testing revealed wild-type BRAF and NRAS. MRI on August 4, 2021, detected a soft-tissue lesion consistent with tumor recurrence, meeting RECIST criteria for progressive disease (PD).

On August 6, 2021, he underwent navigated resection of residual tumor with middle/inferior turbinectomy, maxillary sinus opening, and skull base clearance. From September 20 to December 31, 2021, he received five cycles of pembrolizumab (100 mg q3w). Endoscopic evaluation on December 22, 2021, revealed mucosal swelling; biopsy showed atypical melanocytic infiltration consistent with persistent disease (PD by RECIST). On January 3, 2022, he underwent extended resection followed by seven additional cycles of pembrolizumab (January 17-June 20, 2022).

**Figure 1:**
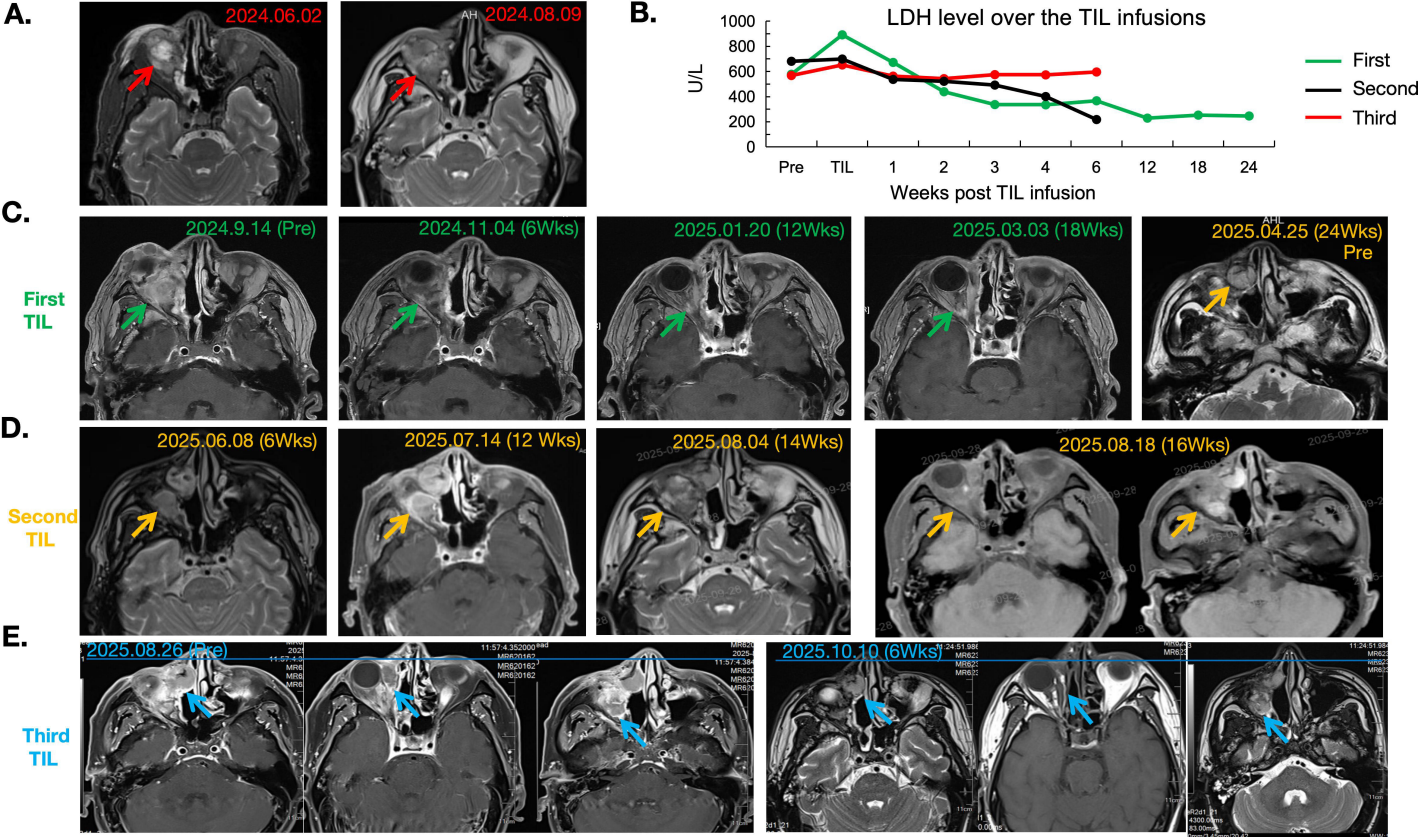
MRI scans of the patient over the course of TIL treatment. **(A)** MRI scans of the patient showing an increased tumor size of 2.4cm prior to enrollment in the TIL trial. **(B)** Serum LDH level alteration over the three TIL infusions, showing consistent changes with decreased tumor burden. **(C)** MRI scans showing the tumor size of the first pre-TIL and post-TIL treatment (2.4cm and 0 cm, respectively), indicating a complete response (CR) according to RECST 1.0 criteria. **(D)** MRI scans showing the tumor size after the second TIL infusion, indicating a short stable disease followed by rapid tumor progression within 2 months. **(E)** The third TIL infusion induced a rapid tumor shrinkage at 6 weeks after TIL treatment, with tumor size decreasing from 6.2cm to 3.0 cm in 6 weeks. LDH, lactate dehydrogenase.

By October 27, 2022, endoscopy revealed a right nasal mass; histopathology confirmed melanoma relapse (PD). On November 8, 2022, a fourth surgery with skull base debridement was performed. Disease recurred again on June 9, 2023 (fifth resection). Thereafter, he received three cycles of nivolumab (1 mg/kg) + ipilimumab (3 mg/kg) from June 26 to August 11, 2023. Despite initial stabilization, the tumor relapsed on September 19, 2023, prompting the sixth surgery and initiation of nivolumab monotherapy (240 mg q2w) from October 12, 2023, to April 19, 2024 (14 cycles). Recurrences necessitated a seventh (November 21, 2023) and eighth (February 21, 2024) surgery. Due to orbital invasion, a ninth resection was performed on May 2, 2024. Two doses of pembrolizumab (May 24 and June 14, 2024) were administered, followed by a tenth resection on June 26, 2024. Combination pembrolizumab (100 mg) and ipilimumab (50 mg) were given on July 19 and 30, 2024. By August 13, 2024, imaging showed extensive recurrence (PD by RECIST) in the nasal cavity and maxillary sinus (**Figure 1A**). Having exhausted standard therapies, including dual ICIs and refusing chemotherapy/radiotherapy, no effective options remained.

Upon learning about an exploratory clinical trial evaluating autologous TIL (LM103) combined with PD-1 blockade in advanced solid tumors, the patient expressed interest. Eligibility screening confirmed inclusion criteria. After comprehensive counseling regarding study design, risks (e.g., lymphodepletion related cytopenia and IL-2–induced cytokine release syndrome), and uncertain benefit, he provided written informed consent. On September 4, 2024, an eleventh surgery was performed to harvest tumor tissue for the first TIL generation. Diagnosis: recurrent T4aNxM1a stage IV nasal melanoma; baseline target lesion: 3.3 cm (**Figure 1C**). Per protocol, he received lymphodepletion (cyclophosphamide 30 mg/kg × 2 days; fludarabine 25 mg/m² × 5 days), followed by infusion of 1.8 × 10¹¹ autologous TILs with a cell viability of 97% on September 23, 2024, and high-dose IL-2 for 6 days. Blood samples were collected for pharmacokinetic and immune monitoring.

He developed transient bone marrow suppression, diarrhea, and mild renal dysfunction—attributed to lymphodepletion and IL-2—which resolved with supportive care. Follow-up assessments on November 5 and December 18, 2024, and January 21 and March 4, 2025, showed progressive reduction in target lesion size from 1.3 cm to 0 cm (**Figure 1C**). Serum lactate dehydrogenase (LDH) levels declined significantly, reflecting reduced tumor burden (**Figure 1B**). RECIST assessment confirmed CR. During this period, he continued receiving pucotenlimab every 21 days.

However, on April 25, 2025, local recurrence was detected (1.8 cm; **Figures 1C, D**). By May 19, 2025, the lesion had grown to 3.4 cm (PD). A twelfth surgery was performed on April 28, 2025, for second TIL production. Second TIL infusion (1.3 × 10¹¹) with a cell viability of 91.8% was administered on May 28, 2025, using the same conditioning and IL-2 regimen. Follow-up on June 8, 2025, showed SD (**Figure 1D**). Three doses of pucotenlimab were given. Nevertheless, tumor increased to 3.9 cm by July 4, 2025 (**Figure 1D**).

**Figure 2:**
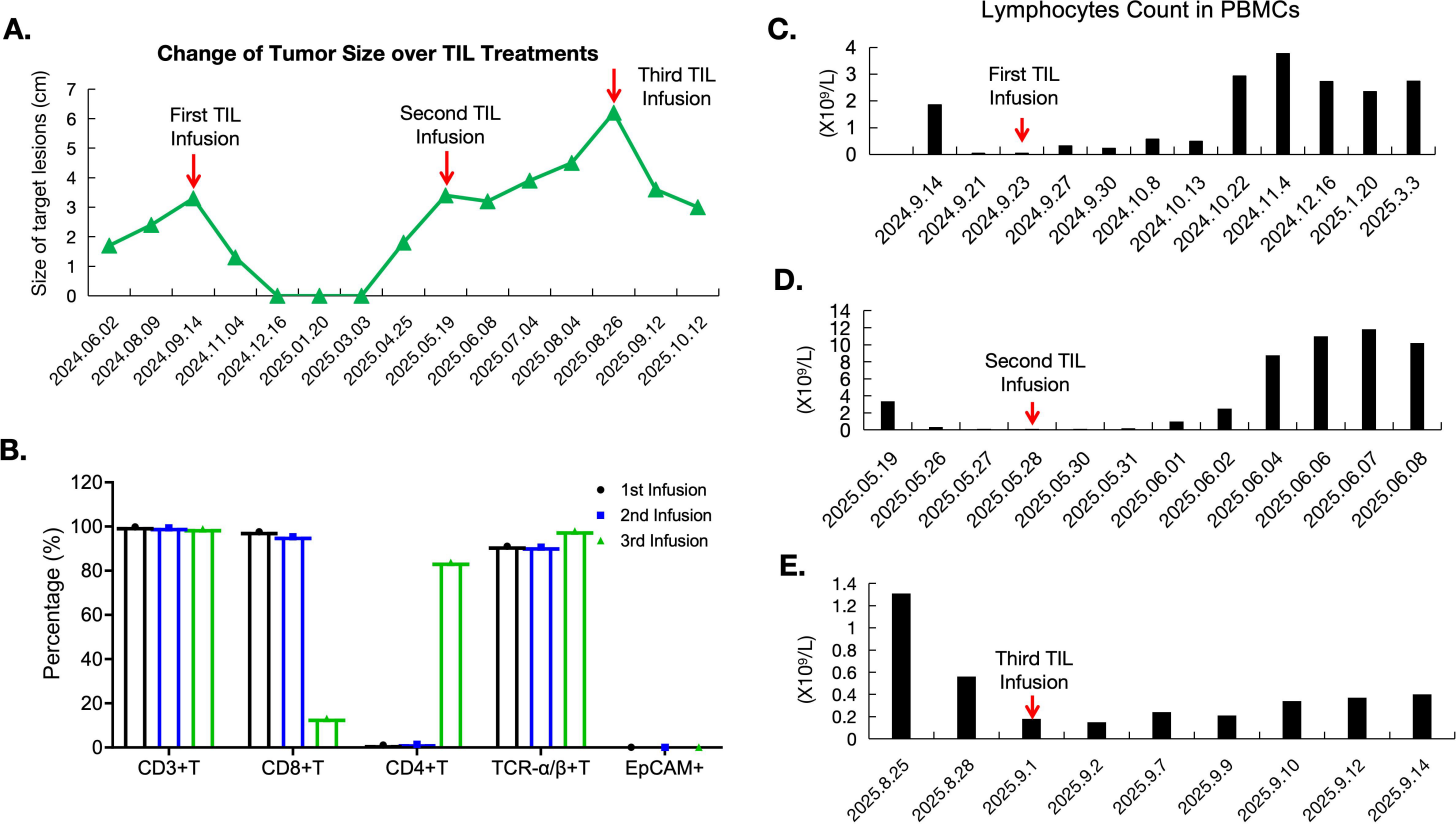
Tumor size change flow chart and peripheral T-cell count over TIL treatment. **(A)** Tumor size alteration over multiple TIL treatments, indicating rapid and repeated tumor shrinkage in two out of three TIL infusions. **(B)** Composition of the three infused TIL products, indicating dominant CD8+TIL cells (97.6% and 95.4%) in the first and second TIL infusions, respectively, but a regnant CD4+TIL of 83.7% in the third TIL infusion, indicting evolution of tumor-infiltrating lymphocytes over the course of the treatment. **(C-E)**. Peripheral T-cell counts during the first, second, and third TIL treatments, showing increased lymphocyte levels within one to four weeks after TIL infusion.

A thirteenth surgery was performed on July 28, 2025, for the third TIL manufacturing. While awaiting product availability, two cycles of ivonescimab (500 mg), a bispecific anti–PD-1/VEGF agent, were administered. However, the tumor rapidly progressed to 6.2 cm by August 19, 2025 (**Figures 1D**, **2A**). The third TIL infusion (5.4 × 10 (10) with a cell viability of 98.9% was delivered on September 2, 2025, with concurrent caduvelilimab (375 mg), a dual anti-PD-1/CTLA-4 agent, to enhance efficacy. Within six weeks, tumor size decreased to 3.6 cm (**Figure 1E**), indicating a PR. Serial tumor measurements across three TIL cycles are illustrated in **Figure 2A**. The complete treatment timeline is detailed in **Supplementary Table 1**. The patient tolerated all three TIL infusions well, with manageable side effects throughout the treatment (**Supplementary Table 2**). The performance status (PS) score of the patient throughout all three TIL treatments remained 0. Manufacturing data for all three infused TIL products are shown in **Supplementary Table 3**.

**Figure 3:**
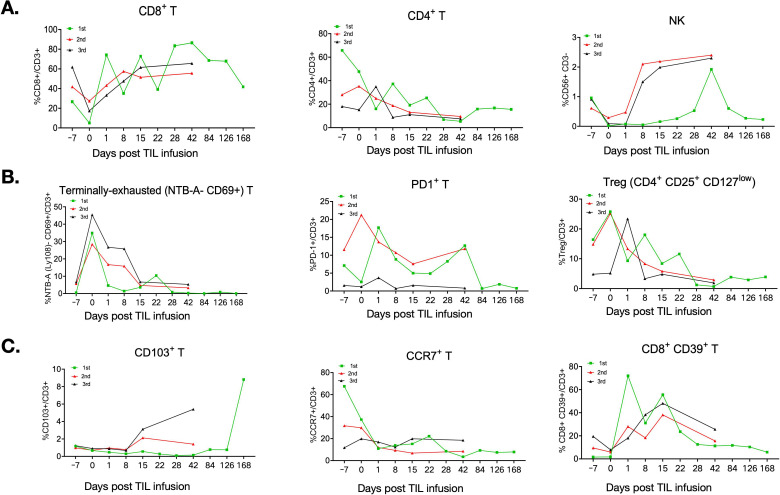
Peripheral blood T-cell sub-typing over the course of the three cycles of TIL treatment. **(A)** Percentages of CD8^+^T, CD4^+^T, and NK cells over the three TIL treatments, showing an increasing trend in CD8^+^T and NK cells and a decrease in CD4^+^T cells after TIL infusion. This CD8+T and CD4+T inversion suggests a systemic immune evolution after TIL treatment. **(B)** Terminally exhausted T cell NTB-A^-^CD69^+^, PD1^+^T cells, and regularly T cells (CD4^+^CD25^+^CD127^low^,Tregs) were generally decreased after TIL treatment, except for a brief increase in PD-1+T cells on day 1 following the first and third TIL infusions. **(C)** Memory T-cell subtype of CD103^+^T increased peripherally after the first and third TIL infusions (which showed objective clinical efficacy), but not after the second TIL infusion (which did not show efficacy). Central memory T cells (Tcm) CCR7^+^T exhibited a general decreasing trend over the three TIL treatments. CD8^+^CD39^+^T cells, an exhausted T-cell subpopulation, were observed with an increasing trend in the first 15 days after TIL infusions and declined gradually thereafter over multiple TIL therapies.

**Figure 4:**
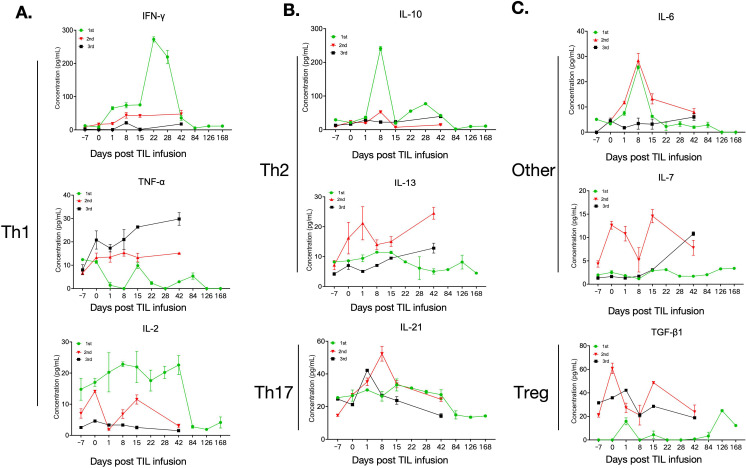
Measurements of cytokine secretion over multiple TIL treatments. **(A)** Th1 cells secreted cytokines, including IFN-γ, TNF-α, and IL-2, were elevated at specific time points after TIL infusion, which is consistent with the clinical response of the patient. Particularly, IFN-γ and IL-2 levels showed a generally higher level in the first TIL infusion compared with the second and third TIL treatments. **(B)** Alterations of Th2 and Th17 cells secreted cytokines, including IL-10, IL-13, and IL21, over the first, second, and third TIL treatments, showing a rising trend during the first three weeks post-infusion but declined thereafter. A transient elevation of the Th2-associated cytokine IL-13 was observed during the second TIL therapy compared with the first and third TIL treatments. **(C)** Cytokines, including TGF-β1, IL-6, and IL-7, generally decreased during the first, second, and third TIL treatments, consistent with clinical responses. The regulary T cell (Treg)-associated cytokine TGF-β showed a higher level on the second TIL infusion compared with the first TIL infusion.

We next performed immune monitoring and biomarker analysis on collected samples. All three TIL products contained >95% CD3^+^ T cells. The first and second products were predominantly CD8^+^ T cells (>90%) with minimal CD4^+^ T cells (<10%). In contrast, the third product comprised only 13% CD8^+^ and 83% CD4^+^ T cells (**Figure 2B**), suggesting evolving tumor microenvironment dynamics. Longitudinal peripheral blood analysis showed increases in total lymphocyte counts following each TIL infusion, temporally correlating with tumor regression (**Figures 2C–E**). Real-time flow cytometry of PBMCs revealed elevated CD8^+^ and NK cell percentages post-infusion during the first, second, and third cycles, while CD4^+^ T cells decreased (**Figure 3A**). Terminal exhausted T cells (NTB-A^-^CD69^+^PD-1^+^) and regulatory T cells (CD4^+^CD25^+^CD127^low^, Tregs) were generally reduced after TIL therapy (**Figure 3B**). Trends toward increased tissue-resident memory T cells (CD103^+^) and regulatory CD8^+^CD39^+^ T cells (particularly at 15 days post-treatment) were also observed (**Figure 3C**). Cytokine profiling revealed transient elevation of Th1-associated cytokines (IFN-γ, TNF-α, and IL-2) following TIL infusion, peaking at time points coinciding with clinical response (**Figure 4A**). Th2 (IL-10 and IL-13) and Th17 (IL-21) cytokines rose during the first three weeks post-infusion but declined thereafter (**Figure 4B**). Expression patterns of TGF-β1, IL-6, and IL-7 mirrored clinical trajectories during the first, second, and third TIL treatments (**Figure 4C**). We further conducted correlation analyses between the progression-free survival (PFS) of the three TILs and the immune parameters, including the percentages of CD3+T, CD4+T, and CD8+T cells in the infused TILs. Additionally, we explored the relationships between PFS and the expressions of main peripheral cytokines, including TNF-γ, TGF-β, and IL13. Results showed no significant correlation between TIL subtypes and PFS (**Supplementary Figure 1A**). However, we observed that the peripheral cytokine expressions of TNF-γ and TGF-β were significantly associated with PFS (**Supplementary Figure 1B**).

## Discussion and literature review

TIL therapy is a form of cellular immunotherapy for cancer based on the patient’s own immune cells. Its core principle is to extract tumor-infiltrating lymphocytes with anti-cancer potential from the patient’s tumor tissue, expand and activate them *in vitro*, and then re-infuse them back into the body to help the immune system more accurately attack cancer cells. It has demonstrated strong anti-tumor potential in multiple advanced solid tumors, including melanoma, cervical cancer, non-small cell lung cancer, head and neck squamous cancer, and is regarded as one of the most important directions in personalized cancer treatment (9, 10). TIL treatment offers outstanding advantages: 1) high specificity: TILs naturally recognize tumor antigens, and persistent cancer-specific T-cell receptors (TCRs) can be generated following successful TIL therapy (11); 2) solid tumor breakthrough: TIL therapy has shown significant efficacy in solid tumors, such as melanoma, achieving long-term remission even after multiple lines of standard therapy (9–11); 3) controllable side effects: TIL therapy carries a lower risk of cytokine storm compared to therapies such as CAR-T (12), and the main adverse reactions are transient fever or fatigue (4–6). However, there are a few limitations to TIL therapy, including high technical requirements such as complex surgical sampling and *in vitro* culture processes (13). Only a limited number of patients were eligible for surgical resection to provide sufficient TIL for expansion, and TIL extracted from initial surgical tumor tissue needs to be cryopreserved for future TIL treatment, especially for those patients who experience rapid recurrence.

To improve efficacy of TIL therapy, several strategies are under investigation. First, optimizing culture technology to develop automated equipment, thereby shortening cell preparation cycles and reducing failure rates. Second, combining TIL with PD-1 inhibitors or targeted drugs to enhance its efficacy. Third, developing universal TIL and “off-the-shelf” TIL through gene editing technology to reduce the cost of individualized preparation, or enhancing its anti-tumor activity. Finally, multiple TIL treatments may be necessary in special circumstances; for instance, when tumors do not significantly shrink after the first treatment, a second TIL treatment can be attempted after evaluation. In addition, for patients who responded to TIL treatment and the relapse, a second or third TIL manufacturing process can be restarted, but new sampling and cell expansion are required. (7).

In this case, the patient received three cycles of TIL infusion and experienced multiple clinical responses, indicating that multiple TIL therapies may be feasible and potentially beneficial, with manageable side effects (**Supplementary Table 2**).The patient derived benefit from the combined therapy of TIL and PD-1 inhibitors for approximately 18 months. This occurred at a critical juncture when standard treatments had failed, and no other active treatment strategies were available. Although the patient eventually developed resistance to the last TIL infusion, the treatment with TIL and PD-1 inhibitors extended survival, improved prognosis, and most importantly, provided an opportunity to access emerging treatments. Furthermore, it has also been reported that repetitive infusions of TIL may lead to durable immunological responses with manageable side effects (14–16). In these previous studies, two to four TIL infusions were administered at intervals ranging from one to three weeks (14–16). Specifically, infusions were carried out at one-week intervals without any pre-treatment for lymphocyte depletion (14–16). However, the key factors affecting the number of TIL treatments, including patient characteristic, sample requirements, treatment interval, dose adjustments of TIL, and IL2 infusion, need to be further explored.

Notably, the second cycle TIL treatment of the patient was not able to induce a significant tumor shrinkage but only a short-term SD, possibly because the infused TILs were not all derived from newly resected tumor tissue (9×10 (10), being partially derived from previously cryopreserved TILs (4×10(10)). This result suggests potential immune escape from previous TIL sample. (7)This hypothesis is supported by the evidence that cytokine profiling revealed transient elevation of Th2-associated cytokine IL-13 and regulary T cell (Treg) associated cytokine TGF-β on second TIL infusion compared with the first TIL (**Figures 4B, C**), implying that the tumor may escape the immune system by producing suppressive cytokines (17). This result also indicates that multiple TIL treatments rely on newly resected tumors each time to obtain TILs for optimal therapeutic effect. Preclinical studies suggest that CD103+ T-cell infiltration predicts response to immunotherapy, and CD103+CD8+ T cells present a tissue-resident memory T-cell (TRM) phenotype and effector function (18, 19). A higher frequency of peripheral blood CD103+CD8+T cells with lower levels of PD-1 expression is associated with favorable outcomes in malignancy, and CD103 on peripheral blood T cells predicts efficacy of immunotherapy of checkpoint blockades in cancer (20, 21). In the present study, we observed that CD103+T cells increased peripherally following the first and third TIL infusions, which showed objective clinical efficacy, but not after the second TIL infusion, implying that increased peripheral CD103+T cells may display a stronger anti-tumor response and enhanced tissue-resident memory T-cell activity (**Figure 3C**). We also observed that peripheral CD8+CD39+T cells increased to higher levels at 15 days after the first and third TIL infusions compared with the second TIL infusion, suggesting that CD8+CD39+T cells may display as a favorable tumor reactivity sub-population over TIL therapy (**Figure 3C**) (22, 23). Furthermore, potential adaptive TIL resistance may also be associated with dysfunctional tumor-reactive TIL clonotypes, loss of antigen-reactive T-cell clonotypes, and absence of intratumoral subclonal neoantigens previously targeted by infused TIL (24, 25). (7) Single-cell profiling and biomarker discovery are needed to uncover the molecular mechanisms of TIL resistance.

Importantly, we observed an increase in the numbers of NK, CD8+T, and CD103+ cells, along with a decrease in the levels of PD1+ and regulatory T cells (Tregs) after TIL therapy (**Figure 3**). An increase in NK cell numbers strengthens the initial tumor immune surveillance capacity, allowing for a prompt response and clearance of tumor cells. The increased number of CD8+T cells can specifically recognize tumor-associated antigens and trigger tumor cell apoptosis through the release of enzymes such as perforin and granzyme. CD103+ cells are associated with tissue-resident memory T cells and can remain in tumor tissue for extended periods, continuously monitoring the tumor microenvironment. In the event of tumor recurrence or the presence of residual tumor cells, they can rapidly initiate an immune response, which is vital for maintaining long-term anti-tumor immunity (26). The increase in the number of CD103+cells lays an immunological foundation for tumor control and regression (18–21, 26). However, the complexity of the tumor microenvironment can significantly impair the effective anti-tumor functions of NK, CD8+T, and CD103+ cells. Tumor cells secrete immunosuppressive cytokines, such as transforming growth factor-β (TGF-β) and interleukin-10 (IL-10), which suppress the activity of NK and CD8+T cells, thereby preventing their normal function (27). Tregs and other immunosuppressive cells also exist within the tumor microenvironment, and can inhibit effector immune cells either through direct contact or the secretion of inhibitory factors. Simultaneously, tumor cells upregulate the expression of immune checkpoint molecules such as programmed death ligand 1 (PD-L1), which binds to programmed death protein 1 (PD-1) on the surface of effector immune cells, transmitting inhibitory signals (27). This leads to the exhaustion of effector immune cell function and the loss of their ability to kill tumor cells (27). Moreover, features of the tumor microenvironment, including hypoxia, low pH, and nutrient deprivation, have a negative impact on the function of NK,CD8+T, and CD103+cells (18–21, 28). Hypoxia reduces metabolic activity and proliferation of immune cells; low pH changes the conformation of receptors on the surface of immune cells, affecting signal transduction and tumor cell recognition; and tumor cells preferentially consume nutrients such as glucose, resulting in insufficient energy supply for immune cells, thereby inhibiting their function (28). Therefore, it may be challenging to overcome the immune suppression of the tumor microenvironment, and TIL therapy combined with PD-1 treatment alone may be insufficient for long-term tumor control. (7) This highlights the importance of the combined application of the triple therapy consisting of TIL, checkpoint inhibitors, and microenvironment modifiers (e.g., anti-vascular endothelial growth factor [VEGF] agents). TSuch therapies provide synergistic effects: TILs supply a large number of immune cells with anti-tumor activity, checkpoint inhibitors block immune checkpoint signaling pathways, and microenvironment modifiers, such as anti-VEGF reagents, inhibit tumor angiogenesis by altering the nutrient supply and metabolic state of the tumor microenvironment, thereby alleviating the effects of hypoxia and nutrient deprivation on immune cells and reducing the secretion of immunosuppressive factors by tumor cells. (7)Through the synergistic role of these three components, the anti-tumor activity of TILs may be maximized, thereby improving tumor regression rates (29).

Furthermore, this study illustrates the transformative potential of multiple autologous TIL infusions in a patient with aggressive, treatment-refractory nasal mucosal melanoma. Despite resistance to multiple ICIs and extensive surgical interventions, the patient achieved repeated objective responses following two of three TIL administrations, each accompanied by detectable systemic immune activation. Notably, the shift in TIL composition—from CD8^+^-dominant in early cycles to CD4^+^-predominant in the third—raises intriguing questions about clonal evolution, antigen escape, and adaptive immune remodeling. The correlation between cytokine dynamics (especially Th1 polarization) and clinical response supports the systemic immunological relevance of infused T cells. While adverse events related to lymphodepletion and IL-2 were expected and manageable, future protocols may benefit from reduced-intensity conditioning or alternative regimens (e.g., IL-15 superagonists and 4-1BB agonists) to improve tolerability. Membrane-bound interleukin-15 (IL-15) has the capacity to facilitate the expansion of TILs, boost anti-tumor activity, and ensure the persistence of CD8+ T cells both *in vivo* and *in vitro*. Moreover, both 4-1BB agonists and IL-15 may help prevent TIL exhaustion (30–32).

This study has several limitations. Firstly, there is an absence of tumor-infiltrating immune profiling and TCR sequencing, which could clarify mechanisms of response and resistance. Secondly, immune monitoring was incomplete during the second TIL cycle. Thirdly, adverse effects associated with high-dose IL-2 administration may limit the broader application of TIL therapy, particularly in patients with abnormal cardio-pulmonary function. In addition, the use of high-dose IL-2 in the treatment of TIL can promote the proliferation and activation of T cells. However, it also raises concerns regarding its potential to induce the expression of PD-1 and T-cell exhaustion (33–36). Previous studies have indicated that high-dose IL-2 may upregulate PD-1 expression on the surface of T cells, potentially through the activation of specific signaling pathways such as the JAK-STAT pathway (35). Activation of this pathway can modify the expression of transcription factors within T cells, consequently increasing the transcription and expression of PD-1 (33–36). Moreover, high-dose IL-2 may induce T-cell exhaustion by pushing T cells into an overactivated state, leading to metabolic disorders and inadequate energy supply, which negatively impacts their normal function (34)^+^. The elevated PD-1 expression induced by high-dose IL-2 is a crucial factor contributing to T-cell exhaustion by transmitting inhibitory signals (33, 34). Fourth, a significant limitation is the use of a mixed TIL source for the second TIL infusion. Although previous studies (37, 38) have demonstrated that cryopreserved TIL cells can be effectively recovered, an 8-month cryopreservation period is relatively long. During this extended interval, TIL cells may lose reactivity and become exhausted (39). Fifth, rapid tumor evolution and tumor genetic heterogeneity over the course of treatment may contribute to treatment resistance (40). Furthermore, there is an absence of TIL and tumor cell co-culture experiments to support the hypothesis that tumor cells may be able to evade immune recognition by TILs. (7) Lastly, there is a lack of molecular characterization by which the TILs play anti-tumor responses *in vivo* and *in vitro*.

Moreover, the applications of TIL therapy are gradually diversifying, with the emergence of TIL combined with immune checkpoint inhibitors, chemotherapy/radiotherapy, genetically modified TIL therapy, and TIL combined with interferon-alpha to enhance efficacy. A phase I clinical trial of TIL therapy combined with a PD-1 antibody in patients with late-stage metastatic lung cancer resistant to PD-1 treatment reported that 20 patients with advanced non-small cell lung cancer who had received four courses of PD-1 antibody treatment were enrolled (41). These patients underwent TIL infusion when disease progression occurred, followed by maintenance treatment with PD-1 antibody. Among the 16 patients who received TIL infusion, 11 experienced tumor regression, including two patients who achieved CR after 18 months and two patients who achieved PR (41). Another clinical trial evaluating the treatment of patients with advanced solid tumors using TIL therapy combined with PD-1 antibody reported ORR of 57.1% in 14 patients with cervical cancer, 60% in 10 patients with metastatic melanoma, and 38.9% in 18 patients with head and neck squamous cell carcinoma (42, 43).

Importantly, several aspects impact TIL and gene-modified TIL-based therapies. Genetically engineered TILs aim to enhance cytotoxic potential, improve tumor homing ability, and reduce T-cell exhaustion (25). Several targets, including TNF-α, IL-2, NFAT.IL-12, CXCR2, CXCR1, and PD-1, have been explored for gene modification of TILs (44, 45). In a phase I/II clinical trial, genetically engineered TILs were modified to produce IL-2 *in vivo* by inserting a retrovirus carrying the human IL-2 gene into TIL, leading to expression of IL-2; these cells were used to treat advanced melanoma. Among seven evaluable patients who received transduced TIL, one patient experienced a partial response associated with *in vivo* persistence of IL-2-transduced TILs in circulating lymphocytes (46, 47). In another gene-modified TIL trial, 33 patients with metastatic melanoma were treated in a cell-dose escalation trial using autologous TILs transduced with a gene encoding a single chain IL-12 driven by a nuclear factor of activated T cells promoter (NFAT.IL12), without giving IL-2 infusion. The administration of 0.001-0.1 X 10(9) NFAT.IL12 transduced TIL in 17 patients resulted in a single objective response (5.9%). While doses in the range of 0.3–3 X 10(9) cells led to 10 out of 16 patients (63%) to achieve objective clinical responses. However, responses tended to be short-lived, and the administered IL-12-producing cells rarely persisted at one month post-treatment (24). All these efforts highlight important strategies to improve the efficacy of TIL and may lead to breakthroughs in the coming years.

Although single-administration TIL monotherapy can achieve a clinical response rate of 30-40% in advanced solid malignancies, more than half of patients do not experience clinical benefit. Given the presence of natural immune inhibitory signaling pathways in certain TIL cell components, such as PD-1/PD-L1 and CTLA-4/B27 pathways, combining TIL therapy with anti-PD-1 or genetically engineered TILs may significantly enhance the therapeutic efficacy of TILs.

## Conclusion

This case emphasizes the possibility and therapeutic potential of multiple TIL treatments combined with immune checkpoint inhibitors in selected patients with anti-PD-1-resistant advanced melanoma. It highlights the importance of personalized, adaptive cellular immunotherapy strategies. Larger prospective studies are warranted to determine the efficacy, optimal scheduling, dosing, and combination regimens for multi-cycle TIL therapy.

## Data Availability

The raw data supporting the conclusions of this article will be made available by the authors, without undue reservation.
