# Supplementary material for: Multiple autologous tumor-infiltrating lymphocyte (LM103 infusion) therapy combined with immune checkpoint inhibitor induces repeated tumor regression in a patient with aggressive mucosal melanoma: a case report and literature review
Source: Front Oncol. 2026 Apr 23;16:1789442. doi: 10.3389/fonc.2026.1789442 (PMC13150752; doi:10.3389/fonc.2026.1789442)
Supplement: Supplementary file 1 [file Table1.docx]

**Supplemental Table 1 Detailed treatment timeline of the patient**

| **Date** | **Treatment** | **Dose** | **Tumor Size (Examine Date)** |
| --- | --- | --- | --- |
| **2021.07.20：Surgical resection and diagnosis as melanoma** | | |  |
| **2021.08.06：Surgical resection** | | |  |
| 2021.09.20 | Pembrolizumab | 100mg | - |
| 2021.10.11 | Pembrolizumab | 100mg | - |
| 2021.11.01 | Pembrolizumab | 100mg | - |
| 2021.11.22 | Pembrolizumab | 100mg | - |
| **2022.01.03：Surgical resection** | | | - |
| 2021.12.13 | Pembrolizumab | 100mg | - |
| 2022.01.17 | Pembrolizumab | 100mg | - |
| 2022.02.21 | Pembrolizumab | 100mg | - |
| 2022.03.14 | Pembrolizumab | 100mg | - |
| 2022.04.04 | Pembrolizumab | 100mg | - |
| 2022.04.25 | Pembrolizumab | 100mg | - |
| 2022.05.23 | Pembrolizumab | 100mg | - |
| 2022.06.20 | Pembrolizumab | 100mg | - |
| **2022.11.08：Surgical resection** | | |  |
| 2022.12.01 | Pembrolizumab | 100mg | - |
| **2023.06.09：Surgical resection** | | |  |
| 2023.06.26 | Nivolumab+Ipilimumab | O:1mg/kg+Y:3mg/kg | - |
| 2023.07.17 | Nivolumab+Ipilimumab | O:1mg/kg+Y:3mg/kg | - |
| 2023.08.11 | Nivolumab+Ipilimumab | O:1mg/kg+Y:3mg/kg | - |
| **2023.09.19：Surgical resection** | | | - |
| 2023.10.12 | Nivolumab | 240mg | - |
| 2023.10.26 | Nivolumab | 240mg | - |
| 2023.11.10 | Nivolumab | 240mg | - |
| 2023.11.22 | Nivolumab | 240mg | - |
| 2023.12.08 | Nivolumab | 240mg | - |
| 2023.12.22 | Nivolumab | 240mg | - |
| 2024.01.19 | Nivolumab | 240mg | - |
| 2024.02.02 | Nivolumab | 240mg | - |
| 2024.02.16 | Nivolumab | 240mg | - |
| **2024.02.21：Surgical resection** | | |  |
| 2024.03.01 | Nivolumab | 240mg | - |
| 2024.03.22 | Nivolumab | 240mg | - |
| 2024.04.05 | Nivolumab | 240mg | - |
| 2024.04.19 | Nivolumab | 240mg | - |
| **2024.05.02：Surgical resection** | | |  |
| 2024.05.24 | Pembrolizumab | 100mg | - |
| 2024.06.14 | Pembrolizumab | 100mg | 1.7 (2024.06.02) |
| **2024.06.26：Surgical resection** | | |  |
| 2024.07.09 | Pembrolizumab+Ipilimumab | K:100mg+Y:50mg | - |
| 2024.07.30 | Pembrolizumab | 100mg | - |
| **2024.08.13：Surgical resection** | | |  |
| 2024.08.28 | Pucotenlimab (Anti PD-1) | 200mg | 2.4 (2024.08.09) |
| **2024.09.04：Surgical resection，TILs production** | | |  |
| **2024.09.23: First TILs therapy（1.8×10^11^）** | | | 3.3（2024.9.14） |
| 2024.10.14 | Pucotenlimab | 200mg | - |
| 2024.11.04 | Pucotenlimab | 200mg | 1.3（2024.11.4） |
| 2024.11.25 | Pucotenlimab | 200mg | - |
| 2024.12.16 | Pucotenlimab | 200mg | 0（2024.12.16） |
| 2025.01.10 | Pucotenlimab | 200mg | 0（2025.1.20） |
| 2025.01.31 | Pucotenlimab | 200mg | - |
| 2025.02.21 | Pucotenlimab | 200mg | - |
| 2025.03.15 | Pucotenlimab | 200mg | 0（2025.3.3） |
| **2025.03.19：Surgical resection** | | |  |
| 2025.04.03 | Pucotenlimab | 200mg | - |
| **2025.04.21：Surgical resection** | | |  |
| 2025.04.23 | Pucotenlimab | 200mg | 1.8（2025.4.25） |
| **2025.04.28：Surgical resection，TILs production** | | |  |
| 2025.05.14 | Pucotenlimab | 200mg | 3.4（2025.5.19） |
| **2025.05.28: Second TILs therapy (1.3X10^11^)** | | |  |
| 2025.06.13 | Pucotenlimab | 200mg | 3.2（2025.6.8） |
| 2025.07.04 | Pucotenlimab | 200mg | 3.9（2025.7.4） |
| **2025.07.21：Surgical resection，TILs production** | | |  |
| 2025.08.06 | Ivonescimab (Anti PD-1 and VEGFR) | 500mg | 4.5（2025.8.4） |
| 2025.08.19 | Camrelizumab (Anti PD-1 and CTLA-4) | 375mg | 6.2（2025.8.26） |
| **2025.09.01: Third TILs therapy（5.4×10^10^）** | | |  |
| 2025.09.11 | Camrelizumab (Anti PD-1 and CTLA-4) | 375mg | 3.6（2025.9.12） |
| 2025.10.10 | Camrelizumab (Anti PD-1 and CTLA-4) | 375mg | 3.0 （2025.10.16） |
