# Supplementary material for: Multiple autologous tumor-infiltrating lymphocyte (LM103 infusion) therapy combined with immune checkpoint inhibitor induces repeated tumor regression in a patient with aggressive mucosal melanoma: a case report and literature review
Source: Front Oncol. 2026 Apr 23;16:1789442. doi: 10.3389/fonc.2026.1789442 (PMC13150752; doi:10.3389/fonc.2026.1789442)
Supplement: Supplementary file 2 [file Table2.docx]

**Supplemental Table 2** Summary of top five adverse events of the patient during each TIL treatment

| **Adverse Events** | **Grade**  **（CTCAE 5.0）** | **Correlative factor** |
| --- | --- | --- |
| **First TIL** | | |
| Fever | 3 | High dose IL2 infusion |
| Thrombocytopenia | 1 | lymphodepleting regimens |
| Edema of both lower limbs | 1 | High dose IL2 infusion |
| Diarrhea | 1 | High dose IL2 infusion |
| Inappetence | 1 | lymphodepleting regimens |
| **Second TIL** | | |
| Fever | 3 | High dose IL2 infusion |
| Leukopenia | 3 | lymphodepleting regimens |
| Inappetence | 1 | lymphodepleting regimens |
| Edema of both lower limbs | 1 | High dose IL2 infusion |
| Diarrhea | 1 | High dose IL2 infusion |
| **Third TIL** | | |
| Fever | 3 | High dose IL2 infusion |
| Leukopenia | 3 | lymphodepleting regimens |
| Abnormal renal function | 2 | High dose IL2 infusion |
| Inappetence | 1 | lymphodepleting regimens |
| Diarrhea | 1 | High dose IL2 infusion |
