# Supplementary material for: Multiple autologous tumor-infiltrating lymphocyte (LM103 infusion) therapy combined with immune checkpoint inhibitor induces repeated tumor regression in a patient with aggressive mucosal melanoma: a case report and literature review
Source: Front Oncol. 2026 Apr 23;16:1789442. doi: 10.3389/fonc.2026.1789442 (PMC13150752; doi:10.3389/fonc.2026.1789442)
Supplement: Supplementary file 3 [file Table3.docx]

**Supplemental Table 3** Manufacturing data of all the three infused TIL products

| **Inspection Items** | **Qualified Range** | **TIL1** | **TIL2** | **TIL3** |
| --- | --- | --- | --- | --- |
| Trait | a white to light yellow milky liquid | a white to light yellow milky liquid | a white to light yellow milky liquid | a white to light yellow milky liquid |
| PH value | 5.5-7.5 | 6.6 | 6.8 | 6.9 |
| Osmolarity (mOsmol/kg) | 800~1600 | 1344 | 1219 | 1285 |
| Cell viability | ≥70% | 97.0% | 91.8% | 98.9% |
| CD3+T% | ≥70% | 99.8% | 98.9% | 97.4% |
| Cell count | ≥5×10^9^ | 1.8×10^11^ | 1.3X10^11^ | 5.4×10^10^ |
| Residual tumor cells (EpCAM+) | Negative | Negative | Negative | Negative |
| Sterility(rapid culture ) | Negative | Negative | Negative | Negative |
| Bacterial endotoxin | <0.5EU/ml | <0.5EU/ml | <0.5EU/ml | <0.5EU/ml |
| Mycoplasma (qPCR) | Negative | Negative | Negative | Negative |
