# Supplementary figures and images for: Multiple autologous tumor-infiltrating lymphocyte (LM103 infusion) therapy combined with immune checkpoint inhibitor induces repeated tumor regression in a patient with aggressive mucosal melanoma: a case report and literature review
Source: Front Oncol. 2026 Apr 23;16:1789442. doi: 10.3389/fonc.2026.1789442 (PMC13150752; doi:10.3389/fonc.2026.1789442)

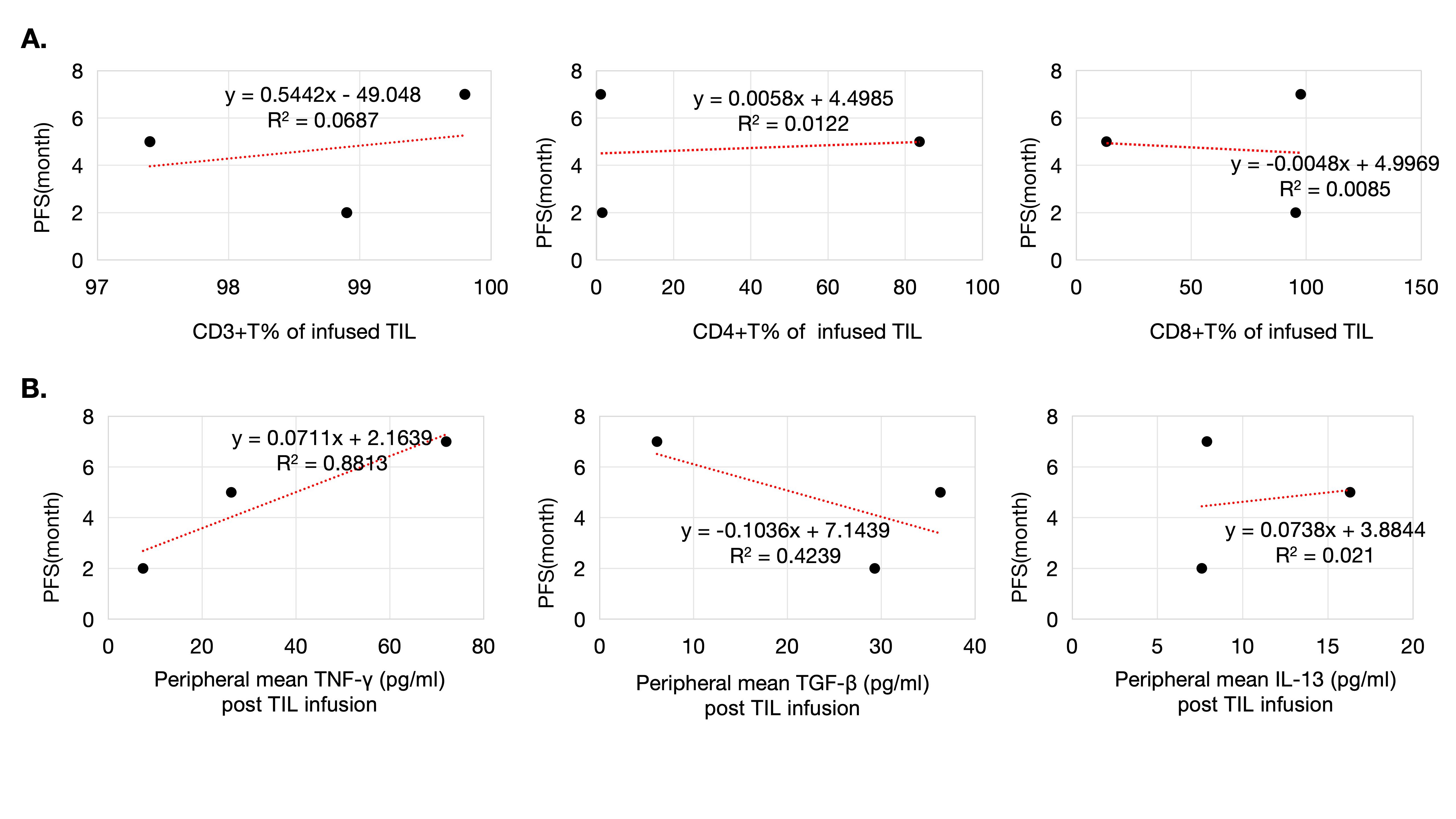

Supplement: Supplementary file 4 [file Image1.jpeg]
